# Amiodarone-Induced Pulmonary Toxicity Presenting as Unilateral Fibrosis: A Case Report

**DOI:** 10.7759/cureus.100729

**Published:** 2026-01-04

**Authors:** Laxman Wagle, Anuj Timshina, Sishir Poudel, Dharana Gelal, Aavash Mishra, Vikas Pathak

**Affiliations:** 1 Internal Medicine, Ascension Saint Agnes Hospital, Baltimore, USA; 2 College of Medicine, B.P. Koirala Institute of Health Sciences, Dharan, NPL; 3 Internal Medicine, Kist Medical College and Teaching Hospital, Lalitpur, NPL; 4 Internal Medicine, TriHealth Good Samaritan Hospital, Cincinnati, USA; 5 Interventional Pulmonology and Critical Care Medicine, Virginia Institute of Lung Diseases, Mechanicsville, USA

**Keywords:** amiodarone-induced pulmonary toxicity, antiarrhythmic drug toxicity, corticosteroid therapy, drug-induced lung injury, high-resolution ct scan, interstitial lung disease, unilateral pulmonary fibrosis

## Abstract

Amiodarone-induced pulmonary toxicity (APT) is a potentially life-threatening complication that most commonly presents as bilateral interstitial lung disease. This case highlights a rare presentation of APT manifesting as unilateral pulmonary fibrosis, posing a diagnostic challenge. An 86-year-old man on long-term amiodarone presented with gradually worsening dyspnea and cough. Chest imaging demonstrated pleural thickening, peripheral opacities, and fibrotic changes confined entirely to the left lung. A broad evaluation, including infectious studies, autoimmune testing, hypersensitivity panels, and bronchoscopy with lavage, did not identify another etiology. Amiodarone was promptly discontinued, and corticosteroid therapy was initiated, resulting in clinical improvement. This case highlights an atypical presentation of APT as unilateral pulmonary fibrosis, which can complicate timely diagnosis due to its asymmetrical radiographic features. Early recognition and withdrawal of amiodarone are essential to limit progression to irreversible fibrosis.

## Introduction

Amiodarone is a class III antiarrhythmic used to manage various forms of arrhythmias. Owing to its high iodine content, lipophilicity, long elimination half-life, and extensive tissue accumulation, amiodarone is associated with multiple organ toxicities, of which pulmonary involvement is among the most serious and potentially life-threatening [1,2]. Ernawati et al. reported ADRAC (Australia) and CDER (USA) data highlighting different patterns of amiodarone side effects [1]. ADRAC found thyroid and skin issues to be the most common, while CDER data showed arrhythmias as the most frequent, with 10% of cases involving lung toxicity such as interstitial lung disease, fibrosis, and pleural effusion. Pulmonary toxicity is among the most feared and serious side effects of amiodarone use, with complications such as progressive interstitial lung disease, irreversible fibrosis, respiratory failure, and death occurring [2]. Interstitial lung disease refers to a group of disorders characterized by inflammation and scarring of the lung parenchyma, which impairs gas exchange [2]. While amiodarone-induced pulmonary toxicity (APT) typically manifests as bilateral injury due to diffuse drug accumulation, rare unilateral presentations occur that may obscure the diagnosis by mimicking alternative etiologies such as infection, malignancy, or focal fibrotic lung disease, thereby delaying appropriate drug withdrawal. This case highlights an uncommon unilateral manifestation of amiodarone-induced pulmonary toxicity and underscores the need for heightened clinical suspicion in atypical presentations.

## Case presentation

An 86-year-old male with a history of systolic heart failure, hypertension, a remote 40-pack-year smoking history, and ventricular tachycardia (on amiodarone 200 mg twice daily for the past six months) presented with worsening dyspnea on exertion and a nonproductive cough lasting one month. He denied fever, chills, rash, joint pains, occupational or environmental exposures, and contact with birds or other animals. There was no personal history of autoimmune or rheumatologic diseases. According to the patient, his chest X-ray 6-8 months back was deemed to be normal.

**Figure 1:**
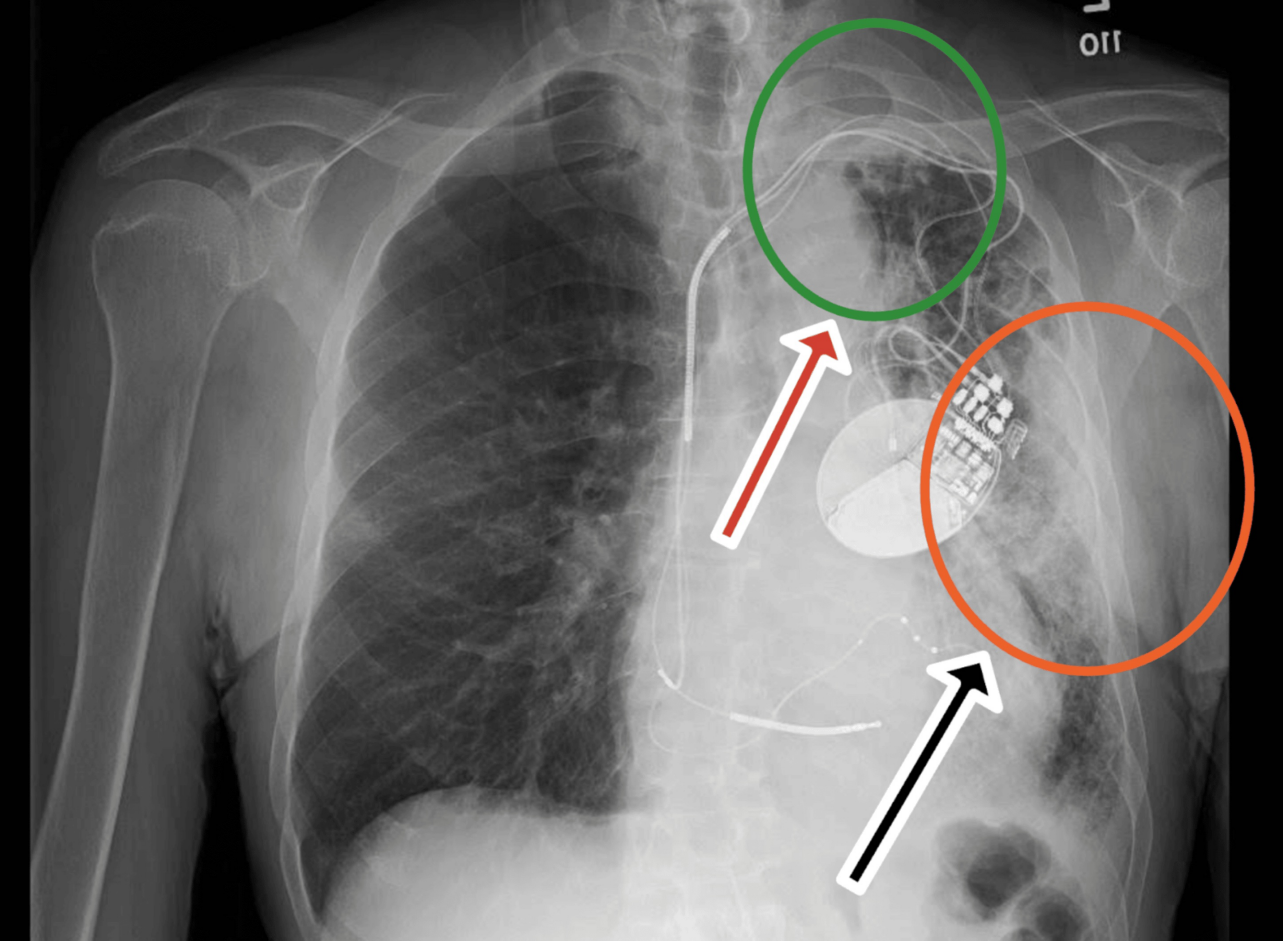
Chest X-ray showing new interstitial thickening and patchy airspace opacities confined to the left upper (red arrow) and lower lobes (black arrow). The marked unilateral distribution is atypical for amiodarone-induced pulmonary toxicity, which more commonly presents with bilateral involvement, and raises diagnostic consideration for alternative focal processes such as infection or malignancy.

**Figure 2:**
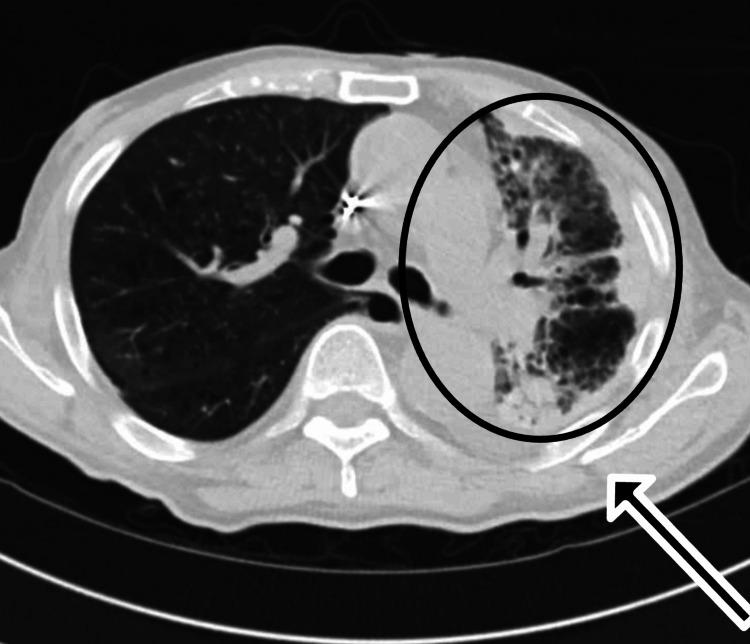
Computed tomography (CT) scan of chest axial view showing extensive pleural thickening, peripheral opacities, and fibrotic changes throughout the left lung. This pronounced asymmetry is unusual for amiodarone-induced pulmonary toxicity and underscores the diagnostic challenge posed by unilateral disease. In the absence of infectious, autoimmune, or malignant etiologies, these findings supported the diagnosis of amiodarone-related lung injury.

On presentation, the patient was afebrile with a blood pressure of 132/68 mmHg, a heart rate of 84 beats/min, a respiratory rate of 20 breaths/min, and an oxygen saturation of 84% on room air, improving to 94% on a 3 L/min nasal cannula. Lung examination revealed left-sided inspiratory crackles without wheezing. Initial laboratory investigations showed no leukocytosis. Chest X-ray revealed new interstitial thickening and patchy airspace opacities confined to the left upper and lower lobes (Figure 1). High-resolution CT (HRCT) of the chest confirmed extensive pleural thickening, peripheral opacities, and fibrotic changes throughout the left lung, along with bilateral emphysematous changes (Figure 2). Laboratory evaluation showed a white blood cell count of 7.8 × 10⁹/L, C-reactive protein of 6.2 mg/L, and erythrocyte sedimentation rate of 38 mm/hr. Autoimmune serologies, including ANA and rheumatoid factor, were negative. These findings aligned with the patient’s clinical symptoms and were not present on prior imaging (the patient reported a normal chest X-ray performed 6-8 months prior to presentation; prior imaging was not available for review), supporting a pathologic rather than incidental process. Pulmonary function testing revealed a restrictive pattern with reduced DLCO, consistent with interstitial lung disease.

The patient was initially suspected of being volume overloaded and was treated with diuretics, but showed no clinical improvement. An extensive workup, including autoimmune and hypersensitivity pneumonitis (HP) panels, was negative. Bronchoscopy with bronchoalveolar lavage (BAL) was performed primarily to exclude infectious and inflammatory mimics of drug-induced lung disease, given the fibrotic pattern observed on imaging. Bronchoalveolar lavage showed normal cellular distribution, with predominantly macrophages (≈85%), lymphocytes 10%, neutrophils 3%, and eosinophils <2%. No lipid-laden macrophages were identified. Bacterial, fungal, mycobacterial, and viral cultures were negative.

Transbronchial biopsy was avoided due to the high risk of possible pneumothorax and low diagnostic yield, given that fibrosis was noted on the HRCT. Amiodarone was subsequently discontinued, and she was started on oral prednisone 40 mg daily, with a planned slow taper over 8-12 weeks. The patient experienced symptomatic improvement and was discharged with plans for outpatient follow-up, including repeat imaging and pulmonary function testing.

Although the patient was lost to follow-up, long-term management would ideally include repeat imaging, pulmonary function testing, and cardiology consultation for the selection of alternative antiarrhythmic therapy.

## Discussion

Amiodarone-related lung involvement is classified into two types: a common, usually asymptomatic lipoid pneumonia known as the “amiodarone effect,” and true pulmonary toxicity, which includes various clinical syndromes such as eosinophilic pneumonia, organizing pneumonia, lung nodules or masses, interstitial lung disease, ARDS, and diffuse alveolar hemorrhage [2]. The mechanisms of APT include direct cytotoxicity or an indirect immune-mediated mechanism [3]. Direct toxicity involves the direct action of amiodarone or its metabolite in lung tissue, causing disruption of cellular membranes, phospholipid accumulation, and production of reactive oxygen species, all of which can cause chronic inflammation and fibrosis. Indirectly, amiodarone may trigger immune hypersensitivity reactions involving lymphocytes, CD8 T-cells, and production of immunoglobulin, causing a cascade of inflammation [3,4]. Mortality from APT varies, ranging from around 9% in patients with chronic pneumonitis to as high as 50% in those who develop acute respiratory distress syndrome (ARDS) [2]. Yamada et al.'s study suggested that the likelihood of developing amiodarone-induced pulmonary toxicity rises progressively with continued use, with estimated cumulative incidences of approximately 4.2% at one year, 7.8% at three years, and 10.6% at five years [5]. Risk factors for amiodarone-induced pulmonary toxicity include advanced age (>60 years), high cumulative and daily doses (> 400 mg/day), duration of therapy, pre-existing lung disease, male gender, recent surgery, and pulmonary angiography [3,6]. The risk is linked to cumulative dose rather than serum drug levels, with toxicity developing months to years after starting therapy [1]. Our patient was an 86-year-old male who had been on amiodarone at a dose of 200 mg twice daily for the past six months. His high-resolution imaging also revealed bilateral emphysematous changes, indicating underlying chronic lung disease. Although his cumulative exposure to amiodarone was relatively low, underlying chronic lung disease, age, and gender remained relevant factors in the development of pulmonary toxicity. Also, he developed progressive dyspnea on exertion and a nonproductive cough beginning approximately one month before admission, suggesting a subacute onset rather than an acute hypersensitivity reaction.

Clinically, APT often presents with nonspecific symptoms. In a study among 28 patients with documented presenting symptoms of amiodarone-induced pulmonary toxicity, the most common initial symptom was dyspnea, observed in 71% of cases. Other reported symptoms included cough in 25%, fever in 21%, and both nausea and generalized weakness or fatigue in 7% of patients [7]. On physical examination, fine inspiratory crackles may be heard. In our case, the patient experienced progressive dyspnea and a nonproductive cough lasting one month, and auscultation revealed inspiratory crackles predominantly on the left side.

Unlike the typical bilateral interstitial involvement seen in amiodarone-induced pulmonary toxicity, unilateral fibrosis can complicate the diagnostic approach by mimicking focal fibrosing interstitial lung diseases (autoimmune causes, radiation injuries), prior infection, aspiration-related injury, or malignancy [1-3]. This asymmetry may lower initial clinical suspicion for drug-induced lung injury and delay appropriate diagnosis and management. Thus, this unilateral presentation, along with the absence of other clinical features, made the diagnosis more challenging.

Diagnosis of amiodarone-induced pulmonary toxicity (APT) is primarily clinical and follows exclusion of other causes, as no single test definitively confirms it. Alternative etiologies should be worked up with appropriate investigations to rule out autoimmune disease, infection, malignancy, and cardiac causes. Chest X-ray is the first-line imaging tool, typically showing diffuse or patchy infiltrates, pleural thickening and/or effusion, and may also reveal reticular, consolidative, or mixed opacities [8,9]. For greater detail, high-resolution computed tomography (HRCT) is more sensitive and can demonstrate ground-glass opacities, septal thickening, and, in more advanced cases, features like honeycombing or traction bronchiectasis. High-attenuation areas in the lungs, liver, and spleen may be seen, reflecting amiodarone deposition in tissue macrophages, a relatively specific finding [10]. Pulmonary function tests (PFTs) often show a restrictive or mixed pattern with reduced diffusion capacity (DLCO), which is often the earliest abnormality [11,12]. Gallium scans, while nonspecific, may help differentiate APT from other conditions like congestive heart failure [13]. Histology from lung biopsy, the diagnostic gold standard, may reveal interstitial pneumonitis, organizing pneumonia, or diffuse alveolar damage with lipid-laden macrophages and lamellar bodies [2,5,14]. However, a biopsy is not always necessary. In most cases, a diagnosis can be made based on clinical and imaging findings after excluding infections, autoimmune conditions, malignancy, and cardiac causes. Infectious etiologies were excluded through negative BAL cultures and absence of systemic symptoms, autoimmune causes were ruled out by negative serologic testing, and malignancy was considered unlikely given imaging characteristics and negative cytology. There was no history of thoracic radiation, pulmonary embolism, or occupational exposure. The exclusion of these competing diagnoses, combined with the temporal association with amiodarone therapy and clinical improvement following drug withdrawal, supports APT as the most plausible etiology. In our case, transbronchial lung biopsy was deferred due to the predominantly fibrotic pattern on HRCT, where diagnostic yield is low, combined with the patient’s advanced age and increased risk of complications such as pneumothorax. Given the supportive clinical context and the potential for biopsy-related morbidity, a noninvasive diagnostic approach was favored.

The primary approach to managing amiodarone-induced pulmonary toxicity (APT) involves immediate discontinuation of the drug and the selection of an alternative antiarrhythmic, ensuring that amiodarone is not reintroduced. In cases where symptoms are moderate to severe or respiratory function continues to decline after stopping the medication, systemic corticosteroids, typically prednisone at a dose of 0.5 to 1 mg/kg per day, may be started, followed by a gradual taper over several months, which has shown a beneficial effect [15-17]. Due to amiodarone’s long half-life and its accumulation in tissues, pulmonary fibrosis may continue to progress even after the drug is stopped. Supportive measures such as supplemental oxygen and enrollment in pulmonary rehabilitation programs can aid in symptom management and functional recovery. Ongoing follow-up with a pulmonologist is essential, with regular clinical evaluations, imaging studies, and pulmonary function testing to monitor improvement or disease progression [2,14,18]. While many patients experience clinical improvement after stopping amiodarone, any fibrosis that has already developed is typically permanent. The overall prognosis is less favorable in patients who present with rapidly progressive disease, extensive lung involvement, or respiratory failure, with mortality reaching up to 10% in severe cases and as high as 50% in those who develop acute respiratory distress syndrome (ARDS) [2].

Unilateral lung involvement in APT is exceedingly uncommon but has been described in a few published reports. Chen et al. [19] reported a case of amiodarone-associated unilateral pneumonitis, while Shaik et al. [20] documented unilateral pulmonary fibrosis attributed to amiodarone exposure. Earlier studies have similarly noted isolated unilateral interstitial changes in patients receiving amiodarone [19-21]. Additionally, imaging literature describes nodular or mass-like patterns of APT, typically peripheral and often involving the upper lobes, believed to arise from localized accumulation of the drug within areas of prior inflammation [9]. Although this mechanism has been primarily proposed to explain nodular variants, a comparable process of focal amiodarone deposition may also underlie unilateral fibrotic involvement. In our case, the exclusive left-sided involvement raises the possibility of localized vulnerability, such as prior undiagnosed inflammatory injury, predisposing the left lung to toxicity. This concept offers a plausible explanation for the asymmetric presentation seen in our patient.

A major limitation of this case is the absence of longitudinal follow-up. The patient was lost to follow-up after hospital discharge, and repeat imaging or pulmonary function testing at 3-6 months was not available. As a result, objective assessment of radiographic resolution, progression of fibrosis, or sustained clinical response to corticosteroid therapy could not be confirmed, limiting diagnostic certainty and long-term outcome evaluation.

## Conclusions

This case demonstrates an atypical unilateral presentation of amiodarone-induced pulmonary toxicity that requires a high index of suspicion for timely diagnosis. While broader literature supports drug discontinuation and corticosteroid therapy in appropriate cases, the lack of follow-up in this patient limits confirmation of treatment response and long-term outcomes. Nevertheless, this report highlights the diagnostic importance of considering drug-induced lung injury in patients with asymmetric pulmonary findings and relevant medication exposure.
